# *Ancylostoma ceylanicum*, novel etiological agent for traveler’s diarrhea—report of four Japanese patients who returned from Southeast Asia and Papua New Guinea

**DOI:** 10.1186/s41182-018-0087-8

**Published:** 2018-03-13

**Authors:** Masahide Yoshikawa, Yukiteru Ouji, Nobuyasu Hirai, Fukumi Nakamura-Uchiyama, Minoru Yamada, Naoki Arizono, Naoaki Akamatsu, Takaharu Yoh, Daisuke Kaya, Toshiya Nakatani, Eiryo Kikuchi, Yuichi Katanami, Kimitoshi Satoh, Ryosuke Maki, Yusuke Miyazato, Yuichiro Oba, Kei Kasahara, Keiichi Mikasa

**Affiliations:** 1 Department of Pathogen, Infection and Immunity, Nara Medical University, Shijo-cho, Kashihara, Nara, 634-8521 Japan; 2 Center for Infectious Diseases, Nara Medical University, Shijo-cho, Kashihara, Nara, Japan; 3 Department of Infectious Diseases, Kyoto Prefectural University of Medicine, Kyoto, 612-8369 Japan; 4 Department of Gastroenterology, Omihachiman Community Medical Center, Omihachiman, Japan; 5 Department of Gastroenterology, Nara Prefectural General Medical Center, Nara, Japan; 6 Department of Infectious Diseases, Nara City Hospital, Nara, Japan; 7 Department of Infectious Diseases, Osaka General Medical Center, Osaka, Japan

**Keywords:** *Ancylostoma ceylanicum*, Soil-transmitted helminth, Imported parasitosis, Southeast Asia, Papua New Guinea, Traveler’s diarrhea, Hookworm diseases

## Abstract

**Background:**

Countries in the Southeast Asia region have a high prevalence of soil-transmitted helminth, such as roundworm, whipworm, and hookworms [*Ancylostoma duodenale*, *Necator americanus*, *Ancylostoma ceylanicum*]. Recent molecular-based surveys have revealed that *A*. *ceylanicum*, a zoonotic hookworm, is likely the second most prevalent hookworm species infecting humans in that part of the world, while others have noted that this infection is an emerging public health risk not only for indigenous people but also for visitors from other countries.

**Case presentation:**

We recently encountered four cases of *A*. *ceylanicum* infection in Japanese individuals who returned from Southeast Asia and Papua New Guinea. Case 1 was a 25-year-old male who stayed in a rainforest in Malaysia for 4 weeks, where he developed abdominal pain and diarrhea in the third week. Eleven adult worms (five males, six females) were expelled after treatment with pyrantel pamoate and identified as *A*. *ceylanicum* based on morphological characteristics and DNA sequences of the mitochondrial cytochrome c oxidase subunit 1 (cox1) gene. Case 2 was a 26-year-old male who spent 2 years as an overseas cooperation volunteer for agriculture in Papua New Guinea. He did not note any symptoms at that time, though eggs were detected in feces samples at a medical check-up examination after returning. Although collection of adult worms was unsuccessful, DNA analysis of the eggs for cox1 and the ribosomal internal transcribed spacer (ITS)-1 and ITS-2 genes demonstrated that they were *A*. *ceylanicum.* Case 3 was a 47-year-old male who spent 1 month in a rural village in Lao People’s Democratic Republic and began suffering from watery diarrhea from the third week. A total of nine adult worms (three males, six females) were collected by endoscopic procedures and following treatment with pyrantel pamoate. Morphological examination and molecular analyses of the cox1 gene showed that they were *A*. *ceylanicum.* Case 4 was a 27-year-old male who participated in group travel to India for 5 days. Three weeks after returning, he developed abdominal pain and diarrhea. Hookworm eggs were found in feces samples and developed into larvae in culture, which were identified as *A*. *ceylanicum* based on molecular analysis of the cox1 gene*.* Eosinophilia was observed in all of the cases prior to treatment.

**Conclusions:**

*A*. *ceylanicum* should be recognized as an important etiologic pathogen of hookworm diseases in travelers to countries in the Southeast Asia and West Pacific Ocean regions.

**Electronic supplementary material:**

The online version of this article (10.1186/s41182-018-0087-8) contains supplementary material, which is available to authorized users.

## Background

Human hookworm infections are mainly attributed to *Necator americanus* and *Ancylostoma duodenale* [1, 2]. *Ancylostoma ceylanicum* can also cause a patent human infection. Although high prevalence rates of *A*. *ceylanicum* in domestic, stray, and community-raised dogs and cats have been reported in Asia, human infection has been largely ignored. Nevertheless, recent molecular-based surveys have revealed that *A*. *ceylanicum* is likely the second most prevalent hookworm species infecting humans in Asia, especially in the Southeastern Asia region [3–9]. Due to the potential for patent enteric infection by this parasite in humans through percutaneous penetration as well as the fecal-oral route, *A*. *ceylanicum* should be recognized as a public health risk, not only for indigenous communities but also for tourists visiting from other countries. We recently encountered four cases of *A*. *ceylanicum* infection in Japanese individuals, each of whom had recently visited Southeast Asia or Papua New Guinea (PNG).

## Case presentation

### Case 1

**Fig. 1:**
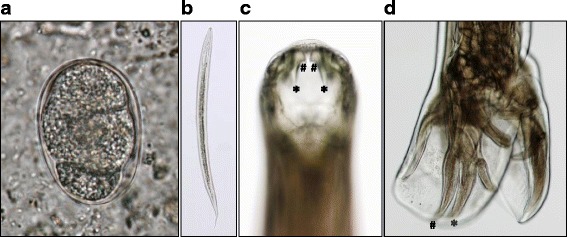
Case 1. **a** Representative four-cell stage egg obtained from feces sample (55.9 × 36.6 μm). **b** A single filariform larva (750 × 25.0 μm), one of many recovered in Harada-Mori culture of feces. **c** Anterior of an adult *Ancylostoma ceylanicum*, showing prominent sets of outer (asterisk) and small inner (number sign) teeth on a cutting plate in the buccal cavity. **d** Posterior of an adult male *Ancylostoma ceylanicum*, showing mediolateral (asterisk) and posterior-lateral (number sign) rays of the copulatory bursa of the running parallel

A 25-year-old male biologist from Japan visited a rainforest in Malaysia in 2009 to collect spiders for study. He developed abdominal pain and diarrhea during the third week. The symptoms did not improve, and he returned to Japan after the fourth week. Because of intermittent abdominal pain and watery diarrhea, the patient visited Kyoto Prefectural University of Medicine, where hookworm eggs were detected in a feces sample prepared by formalin-ethyl ether sedimentation (Fig. 1a) along with an increased number of peripheral eosinophils (3.0 × 10^3^/μL), while neither pathogenic protozoa nor bacteria were identified in fecal samples or culture. Filariform larvae of hookworms were found in a Harada-Mori culture (Fig. 1b). Eleven adult worms, five males and six females, were expelled after treatment with a single dose (10 mg/kg) of pyrantel pamoate and identified as *A*. *ceylanicum* based on morphological characteristics [10, 11], such as one set of prominent outer and one set of much smaller inner teeth on a cutting plate in the mouthpart (Fig. 1c) and parallel mediolateral and posterior-lateral rays of the copulatory bursa in the males (Fig. 1d). Later, DNA was extracted from one of the adult worms and preserved in ethanol solution using a DNeasy Blood & Tissue Kit (QIAGEN) and subjected to a PCR assay specific for the mitochondrial cytochrome c oxidase subunit 1 (cox1) gene. The primer sets used are shown in Additional file 1: Table S1. The cox1 region was analyzed using an ABI Prism 3100-*Avant* Genetic analyzer (Applied Biosystems) and shown to have the *A*. *ceylanicum* sequence (GenBank: accession no. LC271155.1).

### Case 2

A 26-year-old Japanese male spent 2 years as an overseas cooperation volunteer for agriculture in PNG and was examined as part of a medical check-up after returning to Japan in 2014. Although there were no health problems noted, such as dermatitis, fever, and respiratory or abdominal symptoms, during his stay in PNG, laboratory examinations following the return revealed hookworm eggs in feces in direct microscopy observations and increased peripheral eosinophils (1.6 × 10^3^/μL). A Harada-Mori fecal culture failed to obtain fully developed filariform larvae of hookworm. Eggs were obtained by a floatation method with saturated sodium chloride solution, then DNA was extracted and subjected to PCR assays specific for the ribosomal internal transcribed spacer (ITS)-1 and ITS-2 genes and cox1 (Additional file 1: Table S1). Analyses with ITS-1 and ITS-2 (GenBank accession no. LC036567) and cox1 (GenBank accession no. LC036568) sequences identified the eggs as *A*. *ceylanicum.* The patient was treated with pyrantel pamoate, after which eggs became undetectable in feces and the eosinophil count was normalized. Collection of adult worms from feces was unsuccessful. Details of case 2 have been previously reported [12].

### Case 3

**Fig. 2:**
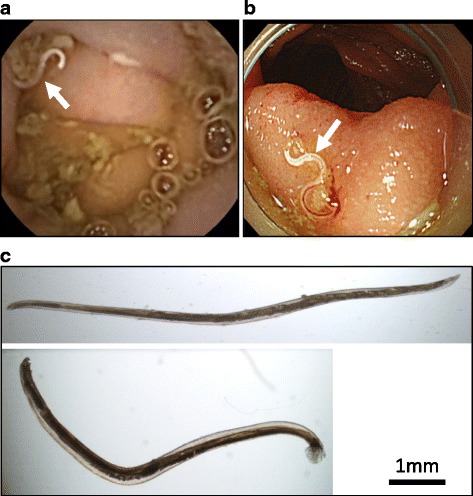
Case 3. **a** Small nematode (arrow) in jejunum shown by capsule endoscopy. **b** Worm sucking blood with head hooked into intestinal mucosa (arrow) shown by double-balloon enteroscopy. **c** One each of female (upper) and male (lower) adult worms obtained in feces after pyrantel pamoate treatment

A 47-year-old male was presented with abdominal pain and watery diarrhea in the fourth week of his stay in a rural village in Lao People’s Democratic Republic. He returned to Japan and visited a local clinic. Laboratory examination showed increased eosinophiles (43%) with normal leukocyte count (7600 cells/μL), but any pathogenic protozoa or bacteria organisms were not detected in fecal samples and culture. The symptoms were improved, but he suffered from intermittent watery diarrhea for a month and referred to Nara Prefectural General Medical Center. Hookworm eggs were found in a feces sample prepared using a formalin-ethyl ether sedimentation method, and filariform larvae of hookworm were successfully obtained in a Harada-Mori fecal culture. Capsule endoscopy findings led us to suspect small nematodes in the jejunum (Fig. 2a). Double-balloon enteroscopy confirmed the presence of worms, including one with its head hooked into the intestinal mucosa and sucking blood (Fig. 2b). A total of three worms (two females, one male) were removed, each of which was identified as *Ancylostoma ceylanicum* based on morphological and genetic examinations using a PCR assay specific for the cox1 gene followed by sequencing (GenBank accession no. LC271184.1). The patient was treated with pyrantel pamoate and six worms (four females, two males) were collected from feces. Figure 2c shows one each of the female and male adult worms. The patient had eaten local food, worn sandals on bare feet, and lived as a local native in a Laotian village for approximately 1 month in August 2015. Details of case 3 have been previously reported [13].

### Case 4

A 27-year-old male attended a 5-day tour to India, including visits to New Delhi, Varanasi, Agra, and Bodhgaya, in September 2016. Fourteen days after returning to Japan, he developed abdominal pain and diarrhea, and the symptoms continued for 2 weeks until initiation of antihelminthic drug treatment. No protozoa or pathogenic bacteria were detected in stool samples or cultures. A direct microcopy examination found hookworm eggs in feces along with increased peripheral eosinophils (7.1 × 10^3^/μL). Larvae were obtained from a Harada-Mori fecal culture and identified as *A*. *ceylanicum* by an examination of the cox1 gene (GenBank accession no. LC271185.1)*.* The patient was treated with albendazole (400 mg/day for 7 days), after which eggs were undetected in feces and consequent normalization of eosinophil count in peripheral blood was seen. Collection of adult worms from feces was not performed. Although the patient had been careful to avoid eating raw vegetables and drinking water without first boiling, he noted that he had lied down and exposed the skin of his upper body to the ground soil in Bodhgaya.

Patient information and clinical features of these four cases are summarized in Table 1. None showed a decreased hemoglobin level in laboratory findings.

**Table 1:** Patient information and clinical and diagnostic features of four Japanese traveler cases of *Ancylostoma ceylanicum* infection

	Case 1	Case 2	Case 3	Case 4
Age (years) sex (F・M)	25・M	26・M	47・M	26・M
Destination	Malaysia	PNG	Laos	India
Symptoms	Abdominal pain	Asymptomatic	Abdominal pain	Abdominal pain
	Watery diarrhea		Watery diarrhea	Watery diarrhea
Hemoglobin (g/dL)	14.5	15.1	13.5	16.1
Eosinophil count (/μL)	3000	1570	20,470^c^	7050
Eggs in feces	Detected	Detected	Detected	Detected
Larvae isolation (Harada-Mori culture)	Succeeded	Failed^a^	Succeeded	Succeeded
Endoscopy	Capsule	Not performed	Capsule	Not performed
			DB enteroscopy	
Adult worms isolated (total, M/F)	11 (5 M/6 F)	0	9 (3 M/6 F)	0
Treatment	Pirantel pamoate	Pirantel pamoate	Pirantel pamoate	Albendazole
GenBank accession number (cox1)	LC271155.1	LC036567.1^b^	LC271184.1	LC271185.1
		LC036568		

^a^Only single larva was obtained. Unfortunately, it had failed to grow fully into a filariform larva

^b^LC036567.1 shows sequences for the gene of 18S rRNA, ITS1, 5.8S rRNA, ITS2, and 28S rRNAva

^c^The value at the visit to Nara Prefectural General Medical center

## Discussion

Although *N*. *americanus* and *A. duodenale* are generally considered to be the most common species related to human hookworm disease [1, 2], a recent molecular-based copro-diagnostic survey revealed that following *N. americanus*, *A*. *ceylanicum* is the second most common in human cases and responsible for between 6 and 23% of total patent hookworm infections in Asia [14]. *A*. *ceylanicum* is commonly found in dogs and cats in Asia, and high rates of infection with that species in domestic, stray, and community-raised dogs and cats have been reported in Malaysia [3], Laos [5], India [15, 16], Thailand [6, 7, 17], Cambodia [8], Vietnam [18], Indonesia [19], and southern China [20]. In addition, infections in dogs as well as humans have also been reported in the Solomon Islands [21, 22] and northern Australia [23–27]. Since *A*. *ceylanicum* causes patent human enteric infections, those living among stray or community-raised dogs and cats have an increased zoonotic risk. On the other hand, human prevalence seems to differ depending on other factors, such as socioeconomic status, general educational attainment in the region, and recognition of the importance of personal hygiene.

Travelers in addition to indigenous individuals are at risk for infection with *A*. *ceylanicum.* We examined four different Japanese individuals who became infected after visiting countries in the Southeast Asia and West Pacific Ocean regions. The patient in case 1 visited Malaysia, where 9.1% of the population is reported to be infected with hookworms, with *A*. *ceylanicum* responsible for 23.4% of those infections [3]. For academic study purposes, he remained for an extended period in a rainforest, where the moist soil is known to be suitable for development and growth of filariform larvae. In case 2, the patient spent 2 years as an agricultural volunteer in PNG, which has a wet equatorial tropical environment and high annual rainfall. Although *N*. *americanus* was previously considered to be the only species present in PNG [28], infection with *A*. *ceylanicum* was reported in two Australian soldiers returning home from service in PNG during World War II [22] (described as *A*. *brasiliensis*, synonymous with *A*. *ceylanicum* at that time). Furthermore, nine service personnel from the Netherlands who had returned from West New Guinea in the early 1960s [29] and an Australian soldier who had stayed in the Solomon Islands, to the east of PNG in Oceania, in 2003 [21], were found to be infected. In the Solomon Islands, a high prevalence of *A*. *ceylanicum* infections in humans was recently noted and those comprised 18.2% of all hookworm disease cases reported in the region [22], suggesting a similarly high prevalence of *A*. *ceylanicum* in PNG. The patient in case 3 lived a reclusive country life in a Laotian rural village, where stray and domesticated dogs and cats were often seen, and ate local food and wore sandals with bare feet. The prevalence of hookworm disease is reported to be 46.3% in Laos, and *A*. *ceylanicum* infection has been shown in 3 (17.6%) of 17 molecularly characterized samples from local individuals with hookworm disease [5]. As for case 4, the patient was mainly in northeast India, which is classified as a humid temperate climate zone. He reported having lied down with the skin of his upper body exposed to the ground soil in Bodhgaya. In a recent study of distinct geographical and climatic locations in India [30], *A*. *ceylanicum* was identified in dogs as either a single or mixed infection with *A*. *caninum* in districts located in a humid temperate or tropical climate zone, though that species was not seen in semiarid or arid mountainous zones. Another study reported a high prevalence of *A*. *ceylanicum* in dogs in Assam, located in northeast India in a humid temperate zone (originally erroneously reported as *A*. *braziliense* [15] and later corrected [16]).

Clinical signs in infected individuals have been reported to range from asymptomatic to heavy symptoms, including anemia, lethargy, and excessive hunger [29]. Three of the present four cases noted major symptoms of abdominal pain and diarrhea, while the other had no symptoms. In observations following experimental infection of human volunteers, abdominal symptoms such as discomfort, pain, flatulence, and diarrhea appeared with eosinophilia within approximately 3 to 4 weeks after infection, regardless of oral or percutaneous route [31, 32]. In other reports that investigated natural infections in travelers [33–35], affected patients had such symptoms as nausea, abdominal pain, watery diarrhea, and bloody diarrhea with eosinophilia. Abdominal pain and mild to severe diarrhea seem to be frequent during the early period following infection. One of the affected patients in a previous report, a French traveler to Myanmar, recalled the development of pruritic erythematous macules on the buttocks after sitting in a public park in Rangoon while wearing short pants [35]. Although the route of infection was not determined in the present cases, a percutaneous route seems to be more likely as compared to an oral route.

A case of neuroretinitis caused by larval migration has also been recently reported [36] and molecular analysis using DNA isolated from intraoperative rinsing fluid determined the larva to be *A*. *ceylanicum.* In that case, a molecular-based technique was the only method available to reach a diagnosis, since the worm was completely destroyed during surgical removal. In the present case 2, eggs from feces were the only evidence obtained. Although morphological examinations of adult and larval worms are important, a molecular approach is of great help for making a diagnosis of hookworm disease including the infecting worm species, especially in cases that lack suitable specimens for morphological diagnosis.

The present cases were only found because of the local network of physicians engaged in parasitic disease treatment and study in Nara, Kyoto, and Osaka prefectures; thus, additional individuals in Japan may have been infected with *A*. *ceylanicum* while visiting countries in the Southeast Asia and West Pacific Ocean regions. We consider that increased attention is needed in regard to *A*. *ceylanicum* infection in patients who are presented with abdominal pain and diarrhea, or even more subtle abdominal symptoms, after traveling to Southeast Asia and West Pacific Ocean countries, as well as northeastern Australia.

## Conclusions

*A*. *ceylanicum* should be recognized as an important etiologic pathogen of hookworm diseases that may infect travelers who visit countries in the Southeast Asia and West Pacific Ocean regions.

## Additional file

Additional file 1: **Table S1.** List of gene-specific primers used for PCR. (PDF 60 kb)

## Abbreviations

*cox1*: Mitochondrial cytochrome c oxidase subunit 1
ITS: Ribosomal internal transcribed spacer
PDR: Lao People’s Democratic Republic
PNG: Papua New Guinea
