# Supplementary material for: Ancylostoma ceylanicum, novel etiological agent for traveler’s diarrhea—report of four Japanese patients who returned from Southeast Asia and Papua New Guinea
Source: Trop Med Health. 2018 Mar 13;46:6. doi: 10.1186/s41182-018-0087-8 (PMC5848582; doi:10.1186/s41182-018-0087-8)
Supplement: Supplementary file 1 — Table S1. List of gene-specific primers used for PCR. (PDF 60 kb) [file 41182_2018_87_MOESM1_ESM.pdf]

Table S1 List of gene-specific primers used for PCR

	Gene	Forward sequence	Reverse sequence
**Case 1, 3, 4**	***COX1***	ttttttgggcatcctgaggtttat	ctaacaacataataagtatcatg
**Case 2**	***COX1***	gtggttttggtaattgaatggtt	taaagaaagaacataatgaaaatg
	ITS*	agaggtgaaattcgtggacc	atatgcttaagttcagcgggt

* the primer set was designed to amplify the gene of 18S rRNA, ITS1, 5.8S rRNA, ITS2 and 28S rRNA.
